# Variance parameter estimation for age at puberty phenotypes under 2 levels of phenotype censorship

**DOI:** 10.3168/jdsc.2022-0218

**Published:** 2022-06-17

**Authors:** M.A. Stephen, S. Meier, M.D. Price, J.E. Pryce, C.R. Burke, C.V.C. Phyn, D.J. Garrick

**Affiliations:** 1 DairyNZ Ltd., 605 Ruakura Road, Hamilton 3240, New Zealand; 2 AL Rae Centre for Genetics and Breeding, Massey University, Ruakura, Hamilton 3214, New Zealand; 3 Agriculture Victoria Research, AgriBio, Centre for AgriBioscience, Bundoora, Victoria 3083, Australia; 4 School of Applied Systems Biology, La Trobe University, Bundoora, Victoria 3083, Australia

## Abstract

Age at puberty (AGEP) is a moderately heritable trait in cattle that may be predictive of an animal's genetic merit for reproductive success later in life. In addition, under some mating strategies (for example, where mating begins before all animals have attained puberty) animals that attain puberty at a relatively young age will also likely conceive earlier than their herd mates, and thus begin their productive life earlier. Unfortunately, AGEP is challenging to measure because animals must be observed over a period of several months. Our objectives for this study were twofold. The first objective was to produce variance components for AGEP. The second objective was to investigate the implications of a simplified phenotyping strategy for AGEP, when the interval between repeated blood plasma progesterone measures was extended from weekly to monthly, increasing the extent of left, interval, and right censoring. We measured AGEP in a closely monitored population of around 500 Holstein-Friesian heifers, born in 2015 and managed under a seasonal, pasture-based dairy system. Animals were blood tested weekly from approximately 240 to 440 d of age and were deemed to have reached puberty when blood plasma progesterone elevation (>1 ng/mL) was detected in 2 of 3 consecutive blood tests (AGEP_Weekly). To simulate a simplified phenotyping strategy based on monthly herd visits (AGEP_Monthly), we selectively disregarded data from all but 3 blood test events, when animals were around 300, 330, and 360 d of age (standard deviation = 14.5 d). The posterior mean of estimated heritabilities for AGEP_Weekly was 0.54, with a 90% credibility interval (90% CRI) of 0.41 to 0.66, whereas it was 0.44 (90% CRI 0.32 to 0.57) for AGEP_Monthly. The correlation between EBVs for AGEP_Weekly and AGEP_Monthly was 0.87 (90% CRI, 0.84 to 0.89). We conclude that in this population, AGEP is a moderately heritable trait. Further, increasing phenotype censorship from weekly to monthly observations would not have altered the main conclusions of this analysis. Our results support the strategic use of censoring to reduce costs and animal ethics considerations associated with collection of puberty phenotypes.

## Highlights

• Age at puberty is moderately heritable in New Zealand Holstein-Friesian cattle.
• The estimated heritability of AGEP is robust to phenotype censorship.
• The AGEP estimated breeding values for animals in this population are robust to phenotype censorship.

## Graphical Abstract

**Figure:**
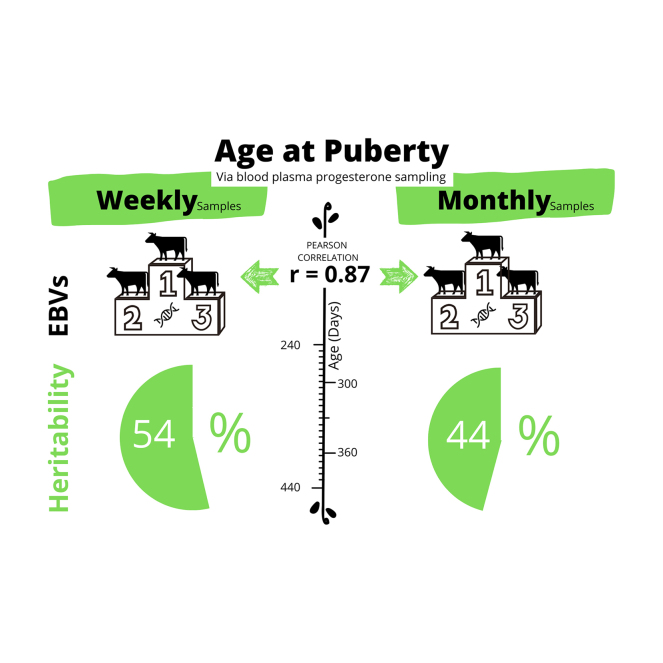
**Summary:** We measured age at puberty (AGEP) in a closely monitored population of approximately 500 Holstein-Friesian cows, born in 2015 and managed under a seasonal, pasture-based dairy system. Animals were blood tested weekly from approximately 240 to 440 days old and were deemed to have reached puberty once blood plasma progesterone (BP4) elevation (>1 ng/mL) was detected in 2 of 3 consecutive blood tests (AGEP_Weekly). To simulate a simplified phenotyping strategy based upon monthly herd visits (AGEP_Monthly), we selectively disregarded data from all but 3 blood test events, when animals were around 300, 330, and 360 days old (SD = 14.5 days). The correlation between estimated breeding values for AGEP_Weekly and AGEP_Monthly was 0.87 with a 90% credibility interval (CRI) of 0.84 to 0.89. The posterior mean of estimated heritabilities for AGEP_Weekly was 0.54 (90% CRI 0.41 to 0.66). Our results support the strategic use of censoring to reduce costs and animal ethics considerations associated with collection of puberty phenotypes.

Reproductive success is a key driver of a dairy cow's lifetime profitability. This is especially true under seasonal, pasture-based grazing systems, where a strictly annual calving pattern dictates that cows must normally resume estrus activity and become pregnant within an 85-d window postcalving. As such, fertility is an important component of the national breeding objective for dairy cattle both in New Zealand (**NZ**) and around the world (Pryce et al., 2014). Unfortunately, many fertility phenotypes that are of direct economic importance have low heritability, and those that are easy to measure are not expressed before the cow is well into its first lactation. The current breeding objective trait for fertility in NZ is derived from the timing of a cow's calving in second lactation relative to the herd's seasonal calving start date. That trait has a heritability of less than 10% (Harris et al., 2006; Bowley et al., 2015), and the phenotype is not expressed until a cow is 3 yr old. Hence, phenotypes that can be measured earlier in an animal's life and can provide a good prediction of an animal's genetic merit for reproductive success would be of high value, particularly if the phenotype has a moderate to high heritability.

Age at puberty (**AGEP**) is a possible candidate trait that meets these criteria. The reported heritabilities of AGEP in cattle range from 0.10 to 0.56 (Smith et al., 1989; Fortes et al., 2012), indicating that it is a moderately heritable trait. Dairy heifers typically reach puberty when they are around 12 mo old, which means AGEP phenotypes are measured substantially earlier than mating- and calving-related phenotypes measured during lactation. There is also a growing body of evidence supporting a genetic relationship between AGEP and subsequent fertility success. For example, Meier et al. (2021) reported that 2 lines of NZ Holstein-Friesian heifers with a divergence of around 1.3 genetic standard deviations in parent average cow fertility had a 28-d phenotypic difference in AGEP. Furthermore, Lefebvre et al. (2021) estimated a genetic correlation of 0.45 (SE ±0.23) between AGEP and postcalving interval to resumption of cyclicity in a population of French Holstein-Normandy cross cattle.

Unfortunately, AGEP is challenging to measure precisely. Animals in a contemporary group can attain puberty over a window of time that spans several months. Numerous and frequent-repeated observations are required to ascertain an animal's precise AGEP. Two common methods for determining an animal's pubertal status involve detecting the presence or absence of an active corpus luteum. The first using ultrasound scanning to visualize the animals ovaries (for example, Fortes et al., 2012), and the second using blood testing for elevated blood plasma progesterone (**BP4**) concentrations (for example, Lefebvre et al., 2021). A third common method involves visually monitoring the animals for signs of estrus, such as mounting behavior (Morris and Amyes, 2005). These indicator phenotypes require a substantial amount of effort and resources to obtain, and daily observations across large cohorts of animals are simply not feasible; however, costs can be constrained by reducing the frequency of observations, and that strategy is common in the literature. The phenotypes analyzed by Fortes et al. (2012) were derived from ultrasonography conducted at approximately monthly intervals, whereas the phenotypes analyzed by Lefebvre et al. (2021) were derived from blood samples taken at 10-d intervals. Although phenotype censoring is a useful strategy for reducing effort and resource requirements, there is an unavoidable trade-off against phenotype accuracy. That said, Donoghue et al. (2004) investigated this trade-off in the context of right-censored fertility phenotypes, where they simulated uncensored, and 12% and 20% right-censored phenotypes. They did not report significant differences in variance parameters and determined that sire EBV rankings were largely consistent across censorship scenarios. Here, we aimed to characterize the heritability of AGEP in a research population of approximately 500 Holstein-Friesian dairy cows (Meier et al., 2021). A second key aim was to determine the implications of left, interval, and right censoring of AGEP on the outcomes of subsequent analysis. Our hypothesis was that phenotype censorship would not meaningfully alter the estimated heritability of AGEP in our population, nor the EBV rankings of animals.

The Ruakura Animal Ethics Committee (Hamilton, New Zealand) approved the study and all manipulations (AE application #13574). We used data collected from a purpose-bred research herd of approximately 500 NZ Holstein-Friesian cows, born in 2015. The population and sampling procedure were described comprehensively by Meier et al. (2021). Briefly, these cattle resulted from planned seasonal matings (where inbreeding coefficients between mating pairs were <12%) designed to generate a herd with extreme divergence in parent average genetic merit for the NZ Fertility Breeding Value (positive line, **POS** +5% EBV; negative line, **NEG** −5% EBV). Milk volume, fat, protein, and liveweight parent average EBVs and proportion of North American Holstein ancestry were constrained to be similar (within 1 SD) in the 2 lines. A total of 67 sires were represented in this population, with 24 and 43 in the POS and NEG fertility groups, respectively. Following rearing, animals were managed on the same farm location in 1 of 4 different herd grazing groups, which were based upon date of birth, while balanced for fertility group.

Animals were blood sampled weekly from approximately 190 kg of BW (~240 d of age) through until they either met the criterion for having attained puberty, or 3 wk after the start of the seasonal breeding period (~440 d of age). Concentrations of BP4 were measured in all these samples, as previously described by Meier et al. (2021).

We used 2 methods to derive an AGEP phenotype using BP4 for every animal. The first method included all available BP4 values, resulting in a weekly testing interval for most animals. Under this method, an animal's AGEP was defined as their age on the day when BP4 concentrations were first observed >1 ng/mL, provided BP4 was also elevated on either of the next 2 blood test days (AGEP_Weekly). If an animal had no measured elevation in BP4 during the study (n = 36), their AGEP was set as their age on the last blood test day plus a 7-d penalty. The mean difference in AGEP_Weekly between the POS and NEG fertility groups was approximately 28 d. That said, there was substantial crossover in the distributions of phenotypes from the 2 groups. The minimum and maximum phenotypes in the POS and NEG groups were 255 to 457 d and 285 to 472 d, respectively. The second, more censored version of this phenotype was derived from the same data, but we selectively disregarded most weekly blood test days to simulate a herd visit testing regimen with only 3 visits at ~30-d intervals (AGEP_Monthly). We chose which 3 blood test days we would include based on the average age of the animals at each visit. We aimed to have roughly even numbers of animals with left- and right-censored phenotypes, and so we selected the second blood test day for when the average age of the animals in each grazing group was ~330 d of age. At this age we would expect roughly 45% of the animals to have attained puberty (Dennis et al., 2018). To produce our AGEP_Monthly phenotype, we defined the AGEP for each animal as their age on the first of the 3 visits that their BP4 concentration was observed >1 ng/mL. If an animal had no measured elevation in BP4 during these visits (n = 299), their AGEP was set as their age on the last of the 3 blood test visits plus a 31-d penalty. This penalty was chosen based on the assumption that most animals would have attained puberty before a fourth herd visit. AGEP_Monthly represents a phenotype with left, interval, and right censorship.

The blood sampling regimen for this trial meant that once an animal had been confirmed as pubertal (that is, the criteria for AGEP_Weekly had been met), future samples from the animal were not quantified for BP4. This meant that due to missing data, some animals that attained puberty during the trial were not observed to have BP4 elevation on any test days that were included to produce the AGEP_Monthly phenotype. In this situation, the animal's AGEP_Monthly phenotype was set as their age on the first (monthly) test that was included after they met the AGEP_Weekly criteria, because if the sampling approach had actually been monthly, these animals would not have been missing and would have been confirmed as postpubertal on this visit. For example, under the AGEP_Monthly scenario, between 13% and 20% of animals were missing on the visit 3 blood test day from each grazing group because they had previously been confirmed as pubertal.

On average, around 10% of animals were also missing from each of the AGEP_Monthly test days for other reasons (that is, they were not sampled on the test day and had not previously met the AGEP_Weekly criteria). These animals did not have the opportunity to be observed as pubertal until at least the following visit, and as such their AGEP_Monthly phenotype may have been inflated by 30 d.

All the animals were genotyped by GenomNZ (AgResearch) using a GeneSeek GGP Bovine 150K SNP array (Illumina). Most other relatives in our genotype database were tested on the Weatherbys Versa 50k SNP array (Weatherbys), so we imputed the genotypes to that content using FImpute software (Sargolzaei et al., 2014). The GeneSeek GGP Bovine 150K SNP array has around 40K SNP in common with the Weatherbys Versa 50k SNP array. For the analyses, we used only the SNP content from the Weatherbys Versa 50k SNP array, disregarding a further 2,120 SNP with a minor allele frequency <1%, leaving some 46,577 SNP.

We fitted a linear SNP effects model to AGEP phenotypes to estimate fixed herd grazing group effects, random SNP effects, and variance parameters. Matrix representation of the linear mixed model equation is

[1] **y** = **Xb** + **Ma** + **e**,

where **y** is a vector of phenotypes (one phenotype per study animal, either AGEP_Weekly or AGEP_Monthly), **b** is a vector of herd grazing group effects, and **a** is a vector of SNP effects. The vector **e** is a vector of residuals corresponding to each of the phenotypes. The incidence matrix **X** relates each phenotype record to relevant fixed effects. The covariate matrix **M** relates each phenotype record to the alleles present at each SNP locus. **M** has a column for each SNP locus, and a row for each phenotype. We ran 2 versions of this model, the first (model 1) included only herd grazing group (4 levels) as a fixed effect. The second (model 2) included herd grazing group and fertility group (2 levels) as fixed effects. Model 2 was included to provide a lower bound for heritability estimation, recognizing that the divergent herd structure could be manifesting as inflated heritabilities in our analysis using model 1 (Price et al., 2017); see below for further details.

A Markov chain Monte Carlo technique was applied using a single site Gibbs sampler to obtain samples from the posterior distributions of variance parameters as well as the fixed and SNP effects. BayesC methodology was used, where Pi (the proportion of SNP loci with 0 effect) was assumed to be 0.99, which meant about 460 SNPs were fit in each sample to explain differences between 525 animals. The Markov chain Monte Carlo comprised 50,000 samples of every unknown parameter, with the first 10,000 samples disregarded as a burn-in. Prior values for genetic and residual variances were 297 d^2^ and 603 d^2^, respectively, for all analyses based on existing NZ data (Dennis et al., 2018). We produced 90% credibility interval (**CRI**) based on thresholds for the 5% (lower bound) and 95% (upper bound) percentiles. We tested our analyses for evidence of nonconvergence using the method described by Geweke (1992). In addition to this diagnostic, we observed trace plots to visually assess the convergence of each parameter.

The extent of re-ranking between EBV produced using the AGEP_Weekly and the AGEP_Monthly phenotypes was quantified using the Pearson correlation coefficient. Correlations included all animals with phenotypes (n = 525). We used a bootstrap method with replacement (Zhu, 1997) to generate the mean and 90% CRI for the correlations between EBVs. These statistics represent a total of 1,000 bootstrap samples.

The heritability and variance components for AGEP_Weekly and AGEP_Monthly phenotypes using both model 1 and model 2 are presented in Table 1. Using either phenotype there was a difference of around 0.10 in the heritabilities estimated using model 1 and model 2. The heritabilities estimated using AGEP_Weekly tended to be about 0.10 higher than those estimated using AGEP_Monthly, but there was a large overlap in 90% CRI for both model 1 and 2. Conversely, the variances themselves were quite different, with higher genetic and residual variances observed when AGEP_Weekly phenotypes were used, compared with AGEP_Monthly phenotypes. This heterogeneous variance across the 2 phenotypes was observed using both model 1 and 2.

**Table 1:** Variance parameters for age of puberty (AGEP) produced using weekly (AGEP_Weekly) or monthly (AGEP_Monthly) sampling^1^

Parameter	Model 1		Model 2	
	AGEP_Weekly	AGEP_Monthly	AGEP_Weekly	AGEP_Monthly
Genetic variance	1,000 (743–1,262)	344 (239–449)	757 (508–1,015)	250 (153–355)
Residual variance	866 (633–1,109)	431 (331–530)	967 (733–1,213)	473 (371–578)
Phenotypic variance	1,866 (1,710–2,034)	775 (706–850)	1,724 (1,571–1,890)	724 (656–796)
Heritability	0.54 (0.41–0.66)	0.44 (0.32–0.57)	0.44 (0.30–0.57)	0.35 (0.22–0.48)

1 Table includes results (with 90% credibility interval in parentheses) from 2 model equations. Model 1 included only herd grazing group as a fixed effect. Model 2 included both herd grazing group and fertility group as fixed effects.

The correlation between EBVs produced from model 1 and model 2 using the AGEP_Weekly phenotypes was 0.90 (90% CRI: 0.90, 0.92). Similarly, the correlation between EBVs produced from model 1 and model 2 using the AGEP_Monthly phenotypes was 0.91 (90% CRI 0.90, 0.92). Within fertility group these across-model correlations were >0.99. The correlation between EBVs produced using either AGEP_Weekly or AGEP_Monthly phenotypes from model 1 was 0.87 (90% CRI: 0.84, 0.89). The correlation between EBVs produced using either AGEP_Weekly or AGEP_Monthly phenotypes from model 2 was 0.84 (90% CRI: 0.81, 0.86). Within each fertility group these across-phenotype correlations ranged from 0.77 to 0.87. Using either model 1 and model 2 the correlations within the NEG fertility group tended to be lower than those within the POS fertility group.

Our AGEP phenotypes were measured in a research herd that consisted of 2 subpopulations with extremely divergent fertility EBVs. This pre-selection on fertility contributed to a divergence of 28 d in the AGEP_Weekly phenotype between the 2 divergent populations (Meier et al., 2021), and a slightly nonnormal distribution in AGEP phenotypes in this research herd. We have analyzed AGEP in a univariate context, and as such, pre-selection on fertility EBVs is not implicitly taken into account. To address this pre-selection, we included a second analysis, using an alternative model equation (model 2) where fertility group is included as a fixed effect (Price et al., 2017). In model 1, herd grazing group was the only fixed effect, whereas in model 2, both herd grazing group and fertility group were fitted as fixed effects. Both models included SNP effects. Under model 1, pre-selection on fertility is ignored, and it is possible that this gives rise to an upward bias in our estimates of genetic variance and heritability of AGEP in this population. Under model 2, pre-selection on fertility is accounted for by omitting comparisons between animals across the 2 fertility groups; however, analysis using model 2 is likely to produce a downward bias in our estimates of the genetic variance and heritability. In this way, the results produced by model 1 and model 2 provide somewhat of an upper (model 1) and lower (model 2) bound for heritability in this pre-selected population. Hence, we estimate that the heritability of AGEP in this population falls between 0.30 (lower 90% CRI under model 2) and 0.66 (upper 90% CRI under model 1) when using weekly BP4 testing. Under increased censorship using AGEP_Monthly phenotypes, these lower and upper bounds are 0.22 and 0.57, respectively. Therefore, in this population, AGEP was a moderately heritable trait, similar to the range of heritabilities reported in other populations (Smith et al., 1989; Fortes et al., 2012). Moreover, we did not observe meaningfully different heritability estimates under increased phenotype censorship by reducing the frequency of BP4 testing or the length of the observation period.

The high correlation between EBVs estimated using AGEP_Weekly phenotypes and those estimated using AGEP_Monthly phenotypes indicated that increasing phenotype censorship did not substantially affect animal EBV rankings. This finding is relevant for animal breeders aiming to apply selection to the AGEP trait in large populations. It is likely that a simplified phenotyping strategy of monthly observations can be implemented, without substantial implications on animal selection decisions.

The variances of the AGEP_Weekly and AGEP_Monthly phenotypes were heterogeneous. This would make it difficult to combine phenotypes that were collected under different phenotyping regimens (for example, where AGEP was measured monthly in one population, and weekly in another). This should not be a problem in a research context, where the same phenotyping strategy is applied to all animals; however, implementation in large-scale animal evaluation schemes may require further work to establish a method for combining censored and uncensored phenotypes in a single analysis. For example, a multitrait approach could be explored.

The main benefits of the AGEP_Monthly phenotyping strategy are the reduced costs, increased practicality, and improved animal ethics considerations associated with measuring the trait. Reducing these barriers to data collection would likely result in an increased number of cows with phenotypes, relative to the number cows that could feasibly be measured for AGEP_Weekly. To capture the value of these additional phenotypes, we would need to manipulate the number of animals contributing phenotypes to each of the AGEP_Weekly and AGEP_Monthly analyses. That is, we would need to reduce the number of animals included in the AGEP_Weekly analysis. Our current data set is too small to test the implications of a relative difference in population size between censorship levels. Our results could be extended either using simulated AGEP phenotypes, or those collected from a larger scale phenotyping initiative. Moreover, collecting AGEP phenotypes for a larger number of animals would be beneficial to investigate the suitability of AGEP as an early predictor trait of subsequent reproductive success. Using a more censored approach to collect AGEP phenotypes less frequently and over a shorter period will enable this research to progress.

We conclude that AGEP is a moderately to highly heritable trait in dairy cattle. If AGEP were to be measured at a large scale, phenotype censorship could provide a useful strategy for reducing costs and logistical challenges associated with phenotype collection, without compromising the integrity of the subsequent analysis.

## References

[bib1] Bowley F.E., Green R.E., Amer P.R., Meier S. (2015). Novel approaches to genetic analysis of fertility traits in New Zealand dairy cattle. J. Dairy Sci..

[bib2] Dennis N.A., Stachowicz K., Visser B., Hely F.S., Berg D.K., Friggens N.C., Amer P.R., Meier S., Burke C.R. (2018). Combining genetic and physiological data to identify predictors of lifetime reproductive success and the effect of selection on these predictors on underlying fertility traits. J. Dairy Sci..

[bib3] Donoghue K.A., Rekaya R., Bertrand J.K. (2004). Comparison of methods for handling censored records in beef fertility data: Simulation study. J. Anim. Sci..

[bib4] Fortes M.R.S., Lehnert S.A., Bolormaa S., Reich C., Fordyce G., Corbet N.J., Whan V., Hawken R.J., Reverter A. (2012). Finding genes for economically important traits: Brahman cattle puberty. Anim. Prod. Sci..

[bib5] Geweke J., Bernado J.M., Berger J.O., David A.P., Smith A.F.M. (1992). Bayesian Statistics.

[bib6] Harris B.L., Pryce J.E., Xu Z.Z., Montgomerie W.A. (2006). Proceedings of the New Zealand Society of Animal Production.

[bib7] Lefebvre R., Larroque H., Barbey S., Gallard Y., Colleau J.J., Laine A.L., Boichard D., Martin P. (2021). Genome-wide association study for age at puberty and resumption of cyclicity in a crossbred dairy cattle population. J. Dairy Sci..

[bib8] Meier S., McNaughton L.R., Handcock R.C., Amer P.R., Beatson P.R., Bryant J.R., Dodds K.G., Spelman R., Roche J.R., Burke C.R. (2021). Heifers with positive genetic merit for fertility traits reach puberty earlier and have a greater pregnancy rate than heifers with negative genetic merit for fertility traits. J. Dairy Sci..

[bib9] Morris C.A., Amyes N.C. (2005). Proceedings of the Association for Advancement of Animal Breeding and Genetics.

[bib10] Price M.D., Camara M.D., Bryant J.R., Meier S., Burke C.R. (2017). Proceedings of the Association for the Advancement of Animal Breeding and Genetics.

[bib11] Pryce J.E., Woolaston R., Berry D.P., Wall E., Winters M., Butler R., Shaffer M. (2014). World trends in dairy cow fertility. 10th World Congr. Genet. Appl. Livest. Prod..

[bib12] Sargolzaei M., Chesnais J.P., Schenkel F.S. (2014). A new approach for efficient genotype imputation using information from relatives. BMC Genomics.

[bib13] Smith B.A., Brinks J.S., Richardson G.V. (1989). Estimation of genetic parameters among reproductive and growth traits in yearling heifers. J. Anim. Sci..

[bib14] Zhu W. (1997). Making bootstrap statistical inferences. Res. Q. Exerc. Sport.

